# The Influence of Fatigue on the Characteristics of Physiological Tremor and Hoffmann Reflex in Young Men

**DOI:** 10.3390/ijerph20043436

**Published:** 2023-02-15

**Authors:** Joanna Mazur-Różycka, Jan Gajewski, Joanna Orysiak, Dariusz Sitkowski, Krzysztof Buśko

**Affiliations:** 1 Department of Ergonomics, Central Institute for Labour Protection–National Research Institute (CIOP-PIB), 00-701 Warsaw, Poland; 2 Faculty of Physical Education, Józef Piłsudski University of Physical Education, 00-968 Warsaw, Poland; 3 Department of Physiology, Institute of Sport-National Research Institute, 01-982 Warsaw, Poland; 4 Department of Anatomy and Biomechanics, Kazimierz Wielki University, 85-091 Bydgoszcz, Poland

**Keywords:** H-reflex, lower extremity tremor, athletes, kayak/canoe ergometer

## Abstract

The aim of the study was to determine the relationship between changes in physiological tremor after exercise and changes in the traction properties of the stretch reflex indirectly assessed using the Hoffmann reflex test. The research involved 19 young men practicing canoe sprint (age 16.4 ± 0.7 years, body mass 74.4 ± 6.7 kg, body height 182.1 ± 4.3 cm, training experience 4.8 ± 1.6 years). During resting tests, Hoffmann reflex measurements were performed from the soleus muscle, physiological tremor of the lower limb, and the blood lactate concentration was determined. Then, a graded test was carried out on the kayak/canoe ergometer. Immediately after the exercise and in the 10th and 25th minute following the exercise, Hoffmann’s reflex of the soleus muscle was measured. The physiological tremor was measured at 5, 15 and 30 min after exercise. Blood lactate concentrations were determined immediately after physiological tremor. Both the parameters of Hoffmann’s reflex and physiological tremor changed significantly after exercise. There were no significant interrelationships between Hoffmann reflex measurements and physiological tremor in resting and post-exercise conditions. No significant correlation was detected between changes in physiological tremor and changes in Hoffmann reflex parameters. It is to be assumed that there is no connection between a stretch reflex and a physiological tremor.

## 1. Introduction

Relatively new methods of measuring central fatigue include measurements of Hoffmann’s reflex [1] and physiological tremor [2,3]. Hoffmann’s reflex is an electrically stimulated reflex analogous to the mechanically induced spinal reflex. The main difference between the H-reflex and the stretch reflex is that the H-reflex bypasses the muscle spindles, which makes it a good tool for estimating the modulation of monosynaptic reflex activity in the spinal cord. However, the H-reflex does not uniquely reflect muscle spindle activity. The maximal amplitude of the H-reflex is rather normalized to the maximal amplitude of the M-wave and is called the H/M ratio. The H/M ratio changes after fatigue. Some authors reported that the H/M ratio decreased after fatigue [4,5]. In most work, however, the respondents undertake the MVC test [4,6,7] or the test on an Incline [8,9]. So far, no work has been conducted on subjects who would experience fatigue after a typical physical exercise.

The definition of tremor most often appearing in the literature describes it as involuntary, rhythmic oscillatory movements of body parts [10]. Tremor is most easily divided into resting (e.g., parkinsonian) and active tremor (e.g., positional, kinematic or physiological). Physiological tremor is defined as “involuntary oscillations of individual body parts of healthy people resulting from the interaction of mechanical and nervous factors” [2]. These factors include the viscoelastic properties of tissues, force fluctuations resulting from the summation of the contraction force of individual motor units, the effect of the stretching reflex, the synchronization of motor unit excitations and the rhythmic activity of the central nervous system. Physical exercise increases the amplitude of physiological tremor. The values of changes in the amplitude and frequency of tremor depend on the type of effort and its duration [11]. Research shows that both strength training [12] and the effort of increasing intensity [2] significantly affect the increase in the amplitude of physiological tremor. Gajewski et al. [2], studying changes in physiological tremor and hormonal responses during high-intensity efforts in a canoeing group, observed that the pronounced bridge of changes in tremor amplitude was visible in the higher frequency range (10–20 Hz). Elevated amplitude values lasted up to 30 min after the end of exercise. In the case of changes in tremor after intense isokinetic effort, a significant decrease in the amplitude of tremor was demonstrated 25 min after its end [13]. It is believed that the changes in tremor amplitude caused by fatigue result from the temporary disturbance of the mechanisms of control in the nervous system [14]. Fuglsang-Fredriksen and Ronager [15] suggest that a decrease in the frequency of stimulation of motor units may be a symptom of increasing central fatigue. As a result of fatigue in the EMG signal spectral, mainly low-frequency components with a frequency of several hertz increase, which reduces the average frequency of the spectral [6]. Changes in the EMG signal in the low frequency range are explained by the synchronization of the stimulation of motor units [16]. Bacher et al. [17] combine these changes with the influence of the stretch reflex, which, as claimed by Avela et al. [18], changes relatively quickly under the influence of fatigue. The combined stimulation of motoneurons decreases due to a decrease in activity in afferents caused by a decrease in the sensitivity of muscle spindles to mechanical stimuli as a result of eccentric contractions. Reduced stiffness of muscle fibers, as well as increased concentration of metabolites in muscles, may also cause a decrease in activity in afferents. The stretch reflex plays an important role in the collection and use of elastic energy in muscles and tendons in the stretch–contraction cycle. Changing the characteristics of this reflex as a result of fatigue has a significant impact on the ability to generate maximum power during movements involving a stretch–contraction cycle.

In the literature, Hoffmann’s tremor and reflexes have not been considered together until now. The connections between these two phenomena with fatigue and stretch stress seem, however, if not obvious, then at least probable. Hoffmann’s reflex is an objective method to determine the characteristics of the reflex for stretching, but the relationship between physiological tremor and stretch reflex is not explicitly presented in the literature. Lakie et al. [3] do not see the relationship between tremor (in the low frequency range) and stretch reflex, while Cresswell and Löscher [19] confirm that tremor changes affect the properties of the traction reflex. Confirmation of the hypothesis about the quantitative relationship of Hoffmann’s reflex and physiological tremor could ultimately explain the genesis of tremor changes and, conversely, it should be used to assess the part of fatigue associated with changes occurring in the nervous system. A positive result of these investigations would give the opportunity to interpret properly the post-exercise changes in tremor. 

The aim of the study was to determine the relationship between changes in physiological tremor after exercise and changes in the traction properties of the reflex indirectly assessed using the Hoffmann reflex test.

## 2. Material and Methods

### 2.1. Participants

The research was conducted with 19 young men (kayak paddlers n = 9, canoe paddlers n = 10), whose characteristics are presented in Table 1. The research was carried out with the consent of the Ethics Committee of the Institute of Sport in Warsaw and the Senate Ethics Committee of Scientific Research of the Józef Piłsudski University of Physical Education in Warsaw. All subjects were informed about the purpose and methods of research and about the possibility of giving up research at every stage. The respondents gave written consent to participate in the project. In the case of minors, written consent for research was expressed by parents or legal guardians. Most of the respondents belonged to the national team of juniors.

### 2.2. Experimental Design

During resting tests, Hoffmann reflex measurements were performed from the soleus muscle, physiological tremor of the lower limb, and the concentration of blood lactate was determined. Then, a graded exercise test was carried out on the kayak/canoe ergometer. Immediately after the exercise and in the 10th and 25th minute following the exercise, Hoffmann’s reflex of the soleus muscle was measured. Since H-reflex measurements took about 3–4 min, physiological tremor was then measured at 5, 15 and 30 min after exercise. Blood lactate concentrations were determined immediately after each effort in the graded test and after each physiological tremor measurement.

### 2.3. Physiological Tremor

The acceleration method was used to study tremor. The waveforms of the tested signals were recorded using the ZPP-3D/BC Acceleration Measurement Kit, JBA Zb. Staniak, Poland. When measuring the tremor of the lower limb, the subjects were in the forefoot position on a two-centimeter elevation with the torso supported, resting the shoulders against the wall. The accelerometer was placed on the front of the tibia. The measurement lasted for 32 s. Sampling was done at a frequency of 200 Hz. The acceleration signal, after analog-to-digital conversion, was subjected to frequency analysis, the aim of which was to obtain the corresponding power spectral density (power spectral density (PSD)) functions. This function describes the distribution of signal variance in the frequency domain. For the PSD estimation, the fast Fourier transform (FFT) procedure was used with the MATLAB R2007a program. In order to average the values of the power spectral density, the Welch procedure was used, which determines the function proportional to the spectral density G(f) (1) of the random signal. The computational procedure requires dividing the sets of N total samples into N_d_ overlapping segments, each with a length of N.

where:

(1) $$i\;=\;1,\ldots ,N/2;\;k\;=\;1,\ldots ,N_{d}.\;G\left(f_{i}\right)=\frac{1}{N_{d}}\overset{N_{d}}{\underset{k=1}{\sum }}G_{k}\left(f_{i}\right)$$

From the course of average power spectral density (PSD) in the frequency domain, the indicators describing the power of low- and high-frequency components of the tremor signal were calculated.

### 2.4. Hoffmann Reflex

The activity of the soleus muscle of the right lower limb was determined by means of EMG electromyography (Digitimer D360, Hertfordshire, UK) using bipolar surface electrodes (Ambu Blu Sensor N, Ag/AgCl, Ballerup, Denmark) spaced approximately 2 cm apart as recommended SENIAM [20]. Before placing the electrodes on the muscle, the skin was shaved, scrubbed and disinfected with alcohol. The impedance between the electrodes does not exceed 5 kΩ. The ground electrode was placed on the head of the fibula bone. In order to induce the H-reflex and the M-wave from the soleus muscle, the tibial nerve in the popliteal fossa was stimulated with a stimulator (Digitimer DS7A stimulator) with a stimulus lasting 1 ms. In order to determine the recruitment curve of the H-reflex and the M-wave, the current was increased by 2 mA every 8 s until the H-reflex disappeared. The current was then increased by approx. 5 mA until the maximum amplitude of the M (Mmax) was reached. During stimulation, the cathode (1.5 × 1.5 cm) was placed in the popliteal region at the stimulation site, while the anode (5 × 8 cm) was placed over the patella. The maximum amplitude values of the H (Hmax) and M (Mmax) reflexes from which the Hmax/Mmax ratio was calculated were derived from the recruitment curve. In the post-exercise studies, the intensity of the current needed to induce the maximum amplitude of the H-reflex and M-wave and the latency time that was determined from the stimulus artifact until the M-wave was cut were then analyzed, followed by the Hoffmann reflex with the baseline. The recruitment curves were plotted for each of the subjects both in resting conditions and after the effort. During the measurement of Hoffmann’s reflex in the soleus muscle, the subjects were in the fore-lying position on the couch. In order to maintain a neutral, horizontal position, the subject had their face placed in a special recess in the couch. Upper limbs were arranged symmetrically, parallel to the trunk line.

### 2.5. Graded Exercise Test Conducted on the Ergometer

The graded test was performed on the air-braked Dansprint ergometer, I Bergman A/S (version for canoeists or kayakers) at an aperture regulating open resistance in kayak paddlers on item 5, and for canoe paddlers on item 7. During the measurement on the kayak ergometer for a group of kayak paddlers, the competitors were in a simple force. Kayaks were equipped with footrests. Kayak paddlers based their feet on them at an angle of about 45° [21]. In the canoe paddling technique, the competitor kneeled on one lower limb with the foot against the footrest (the thigh and lower leg form an obtuse angle), while the other low extremity was leaning forward on the bottom of the canoe. Kayakers and canoeists made a relatively large quasi-isometric effort with the lower limbs. Each stroke was required to develop the force exerted by the lower limb on the footrest in order to balance the force of the stroke and its yawing moment.

The graded test consisted of five, four-minute efforts, separated by one-minute breaks. The relative power (relative to body weight) that the athletes were supposed to maintain in their subsequent efforts was 0.50 for kayakers; 0.95, 1.40, 1.85 and 2.30 W kg^−1^; and for canoeists 0.4, 0.7, 1.0, 1.3 and 1.6 W kg^−1^. The intensity of the fourth measurement would exceed the anaerobic threshold (corresponding to 4 mmol/L lactate).

### 2.6. Blood Lactate Analysis

In order to determine the blood lactate concentration after each effort in the graded test and at 5, 15 and 30 min after the end of the graded test, a Super GL-2 analyzer Dr. Müller was used. Blood samples, 20 μL of capillary blood was obtained from the ear lobe. 

### 2.7. Statistical Analysis

The analysis of the data was carried out using Statistica 12.0 (StatSoft TIBCO Software Inc., Palo Alto, CA, USA, 2017). Normality of the variables’ distributions was confirmed by the Shapiro–Wilk test. Sphericity of the data was assessed using the Mauchly test. The comparison of indicators during subsequent measurements was made using the analysis of variance for repeated measurements. If any differences were detected, Fisher’s post-hoc (LSD) test was used for further analysis. The Spearman or Pearson correlation was used to study the relationship between parameters (in the case of confirmed normality of distribution). The significance level α = 0.05 was assumed.

## 3. Results

There were no significant differences in anthropometric variables between kayak paddlers and canoe paddlers. In addition, no interaction between the repeat factor and the group (kayak and canoe) for the Hoffmann reflex parameters (i.e., Hmax: F_3,51_ = 0.59, *p* = 0.62, Hmax/Mmax: F_3,51_ = 1.27, *p* = 0.30) was detected. Therefore, the studied groups were analyzed together.

**Figure 1:**
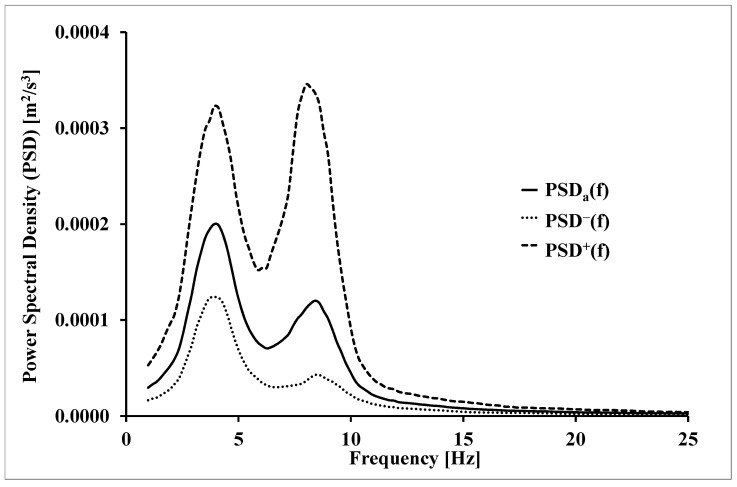
Mean (±SD) waveforms of the spectral density function of the physiological tremor power of lower extremities.

The average waveforms of the spectral density function of the physiological tremor power were similar in shape to all subjects. They showed similar proportions of individual components and the correspondence of frequencies for which maxima occur (Figure 1).

The average waveforms of the power density function along with the waveforms corresponding to the standard deviations were calculated according to the following formulas:

(2) $${\mathrm{PSD}}_{a}\left(f\right)=\exp\left(\frac{1}{n}\overset{n}{\underset{i=1}{\sum }}{\mathrm{lnPSD}}_{i}\left(f\right)\right)$$

(3) $$\mathrm{PSD}\pm \left(f\right)=\exp\left({\mathrm{lnPSD}}_{a}\left(f\right)\pm \mathrm{SD}\left(\mathrm{lnPSD}\left(f\right)\right)\right)$$

where:

n = number of subjects;

i = 1, 2,…n;

SD(lnPSD(f))–standard deviation lnPSD for frequency f.

Due to the apparent skewness of the distributions of the individual components of the tremor, the logarithms of the power spectral density function were further analyzed.

Changes in tremor versus quiescent results were evaluated in the frequency domain by means of the t(f) function, calculated for the entire study group as the value of Student’s t-statistic.

The following formula was used:

(4) $$t\left(f\right)=\frac{{\mathrm{lnPSD}}_{i}\left(f\right)-{\;\mathrm{lnPSD}}_{0}\left(f\right)}{s_{\Delta }}\sqrt{n}$$

where:

PSDi(f)—power density component for frequency f in measurement i = 1, 2, 3;

s_Δ_(f)—standard deviation of the lnPSD differences for frequency f;

n—number of subjects.

**Figure 2:**
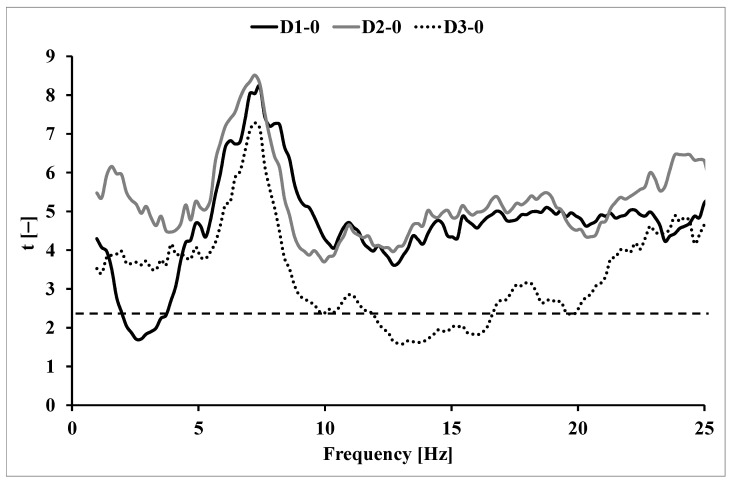
The course of function t(f) for illustrating the significance of tremor power increments relative to the output measurement after a step test; D1–0 for measurement 5 min after exercise, D2–0 for measurement 15 min after exercise, D3–0 for measurement 30 min after the effort; critical t-value (2.10) is represented by dashed line.

The critical value t for 18 degrees of freedom is 2.10 (Figure 2). The critical value on the graph is marked with a dashed line. The course of the function t(f) for the tremor signal after the exercise test in relation to the resting measurement is shown in Figure 2.

Due to the course of the spectral function of the physiological tremor power, the results in the frequency range of 2–5 Hz and 6–10 Hz were extracted for further analysis. Due to the course of the t(f) function of post-exercise measurements in relation to the quiescent measurement, further analyses also included values in the frequency range of 15–25 Hz.

Table 2 presents averages with standard deviations of L indices (logarithmic tremor amplitude index for frequencies in the ranges 2–5 Hz, 6–10 Hz and 15–25 Hz), L_2–5_, L_6–10_, L_15–25_ and frequency fz on the range corresponding to the maxima of the tremor signal (2–5 Hz, 6–10 Hz and 15–25 Hz) f_2–5_, f_6–10_ and f_mean_ for the lower limb under resting conditions and in subsequent measurements after exercise.

By analyzing the indices L_2–5_ (F_3,54_ = 6.35, *p* < 0.001), L_6–10_ (F_3,54_ = 27.50, *p* < 0.001) and L_15–25_ (F_3,54_ = 32.40; *p* < 0.001) were shown to change significantly during subsequent measurements. There were no statistically significant differences between the frequencies of 2–5 Hz (f_2–5_) of the lower limb (F_3,54_ = 1.39, *p* = 0.25). The average frequency (f_mean_) (F_3,54_ = 21.73; *p* < 0.001) changed significantly in subsequent measurements.

The maximum amplitude of the M-wave (Mmax) did not change significantly in the subsequent measurements (F_3,54_ = 1.53, *p* = 0.21), while the maximum amplitude of the Hoffmann reflex (Hmax) (F_3,54_ = 16.05, *p* < 0.001) changed, decreasing immediately after exercise, then increasing; however, even after 25 min from the end of the effort, it did not return to resting values. The ratio of the H-reflex to the M-wave also changed significantly in subsequent measurements (F_3,54_ = 18.20, *p* < 0.001), reaching the lowest value in the measurement immediately after the end of exercise (Table 3).

No significant interdependencies were observed for Hoffmann reflex measurements (Hmax, Mmax, Hmax/Mmax ratio) with physiological tremor (L_2–5_, L_6–10_, L_15–25_, f_2–5_, f_6–10_, f_mean_). No significant correlation was detected between changes in physiological tremor and changes in Hoffmann reflex parameters.

The highest concentration of blood lactate was observed 5 min after the end of exercise, followed by a decrease in the concentration of the parameter examined (Table 4). Negative correlation of blood lactate with L_2–5_ indicator for lower limb (r = −0.463) was observed. There was no significant dependency between the other parameters. 

On the tested subjects, the heart rate after warming up was 121 ± 17 beats per min (bpm) and after exercise 194 ± 5 bpm. A significant correlation was observed (r = 0.527) between the post-exercise heart rate and the physiological tremor of the lower limb in the higher frequency range (6–10 Hz).

## 4. Discussion

Canoeing is a discipline where comprehensive trunk coordination with upper and lower limbs is required [22]. The research indicates a significant influence of the calf muscles (gastrocnemius muscle) on the generation of paddling power [23]; therefore, studies on the soleus muscle are justified. The transmission of the drive to the boat is associated with an intense effort of the lower limbs, in which the triceps muscle of the calf plays an important role in both competitions. In all subjects, we managed to plot the recruitment curve of the H-wave and M-wave reflexes and obtain the power spectral density functions with the maxima occurring around 3 and 10 Hz in all measurements (resting and after exercise). First, we tried to detect the relationship between physiological tremor and the H-reflex in resting conditions. The literature so far has not considered these phenomena together. Tremor itself is not fully understood and still raises a lot of controversy. For example, it is not known whether it is the result of the predominance of central mechanisms [24] or peripheral mechanisms [25]. Cresswell and Löscher [19] suggest that post-exercise tremor changes affect the properties of the stretch reflex. Conversely, Lakie et al. [3] do not see the relationship between tremor (in the low frequency range) and traction stretch. In addition, in our own studies, no correlation was found between the resting parameters of the Hoffmann reflex and the physiological tremor of the lower limb. For the lower limb, the correlation between the examined parameters was also not detected in post-exercise measurements. The research confirmed the significant effect of physical exercise on the Hoffmann reflex and physiological tremor of the lower limb. The highest increase in tremor amplitude was observed 5 min after the end of exercise. It applies to tremor in the higher range (6–10 Hz and 15–25 Hz) frequencies. For frequencies in the range of 15–20 Hz, the increase is also observed 15 min after the end of the exercise. The parameters of Hoffmann’s reflex also change significantly under the influence of physical exercise performed by kayakers. There were no changes in the M-wave amplitude in successive post-exercise measurements in relation to the resting measurement, which is consistent with some literature reports [26,27]. In the literature, however, one can find works in which physical effort with long duration and low contraction force affects the amplitude of the M-wave [28]. There is a decrease in amplitude immediately after exercise and a return to resting values as early as 10 min after the end of exercise [29]. Changes in the amplitude of Hoffmann’s reflex after various efforts or activities are also widely discussed in the literature [30]. It has been shown that a 30 min walk on the treadmill causes a brief decrease in the amplitude of the H-reflex in healthy adults [31]. In addition, Phadke et al. [32] observed the effect of a 20 min gait on the decline in the amplitude of the H-reflex under presynaptic inhibition. Studies indicate that the amplitude of the H-reflex may return to resting values even a minute after the end of the effort on the cycloergometer [33], which does not confirm the results of own studies, showing that the reduction in the amplitude of the H-reflex occurred even 10 min after the end of exercise. The Hmax/Mmax ratio was significantly changed in accordance with changes in the Hoffmann reflex.

Smaller increases in the amplitude of physiological tremor are accompanied by high lactate concentration (about 13.33 ± 2.41 mmol∙L^−1^), which was observed after exercise [2]. In our study, the lactate concentration determined directly after a graded exercise test was on average 6.27 ± 1.73 mmol∙L^−1^. Negative correlations between the amplitude of tremor in the low frequency range (2–5 Hz) and the concentration of blood lactate were observed. There was no correlation between lactate concentration and other parameters of tremor and Hoffmann reflex. Gajewski et al. [2] suggest that the higher the concentration of blood lactate after exercise, the later the return of the amplitude of the physiological tremor to resting values. Such dependence may be related to the previously mentioned phenomenon of the “fatigue paradox”. The relatively high heart rate of the subjects also indicates the high intensity of effort. The demonstrated positive correlation of the heart rate with the increase in tremor of the lower limb in the frequency range of 6–10 Hz is expected and may be another confirmation that physiological tremor is a good indicator of the quantitative assessment of fatigue.

Despite the fact that it was possible to demonstrate the effect of physical exercise on both the Hoffmann reflex and physiological tremor parameters, no convincing relationship was found between the studied phenomena. It should be assumed that the thesis by Vernooij et al. [34] about the lack of connection between the stretch reflex and physiological tremor might be true.

## 5. Conclusions

Hoffmann’s reflex and physiological tremor changed significantly after exercise. There were no significant interrelationships between Hoffmann reflex measurements and physiological tremor in resting and post-exercise conditions. No significant correlation was detected between changes in physiological tremor and changes in Hoffmann reflex parameters. It is to be assumed that there is no connection between a stretch reflex and a physiological tremor.

## Figures and Tables

**Table 1:** Descriptive characteristics for all participants (mean ± SD).

	Age (Year)	Body Mass (kg)	Body Height (cm)	Training Experience (Year)
Kayak (n = 10)	16.3 ± 0.7	76.8 ± 6.2	183.6 ± 4.0	4.5 ± 1.6
Canoe (n = 9)	16.4 ± 0.8	72.2 ± 6.5	180.8 ± 4.4	5.0 ± 1.7
All (n = 19)	16.4 ± 0.7	74.4 ± 6.7	182.1 ± 4.3	4.8 ± 1.6

**Table 2:** Mean (±SD) index of L_2–5_, L_6–10_, L_15–25_ and frequency f_2–5_, f_6–10_ and f_mean_, obtained in subsequent measurements (lower extremities).

	Pre-Test	5′ Post-Test	15′ Post-Test	30′ Post-Test
L_2–5_ (–)	−8.95 ± 0.49	−8.68 ± 0.85	−9.15 ± 0.64 ^b^	−9.25 ± 0.64 ^ab^
L_6–10_ (–)	−9.37 ± 0.83	−8.16 ± 1.28 ^a^	−9.04 ± 1.10 ^b^	−9.63 ± 0.86 ^bc^
L_15–25_ (–)	−12.37 ± 0.50	−11.48 ± 0.86 ^a^	−12.08 ± 0.59 ^ab^	−12.44 ± 0.47 ^bc^
f_2–5_ (Hz)	3.66 ± 0.11	3.60 ± 0.13	3.60 ± 0.14	3.63 ± 0.12
f_6–10_ (Hz)	7.93 ± 0.36	8.45 ± 0.35 ^a^	8.19 ± 0.39 ^ab^	7.93 ± 0.30 ^bc^
f_mean_ (Hz)	6.62 ± 0.82	7.9 ± 0.94 ^a^	7.36 ± 1.08 ^ab^	6.81 ± 1.04 ^bc^

^a^ = significantly different compared to the measurement before test, *p* < 0.05; ^b^ significantly different compared to the measurement 5 min post-test, *p* < 0.05; ^c^ significantly different compared to the measurement 15 min post-test, *p* < 0.05.

**Table 3:** The mean (±SD) of the maximum amplitude of the Hoffmann reflex (Hmax), the amplitude of the M-wave (Mmax) and the ratio of the maximum values Hmax and Mmax (Hmax/Mmax) for measurements before exercise, immediately after exercise and in 10 and 25 min after exercise (n = 19).

	Pre-Test	Immediately Post-Test	10′ Post-TeST	25′ Post-Test
Mmax (mV)	5.18 ± 1.90	5.09 ± 1.81	5.06 ± 1.71	5.00 ± 1.87
Hmax (mV)	2.93 ± 1.76	2.27 ± 1.69 ^a^	2.45 ± 1.55 ^a^	2.60 ± 1.67 ^ab^
Hmax/Mmax (–)	0.56 ± 0.20	0.43 ± 0.22 ^a^	0.48 ± 0.22 ^ab^	0.51 ± 0.22 ^ab^

^a^ = significantly different compared to the measurement before test, *p* < 0.05; ^b^ = significantly different compared to the measurement immediately post-test, *p* < 0.05.

**Table 4:** Mean (±SD) concentration of blood lactate (LA) obtained in subsequent measurements after end of graded exercise test.

	Pre-Test	5′ Post-Test	15′ Post-Test	30′ Post-Test
LA (mmol/L)	1.82 ± 0.41	6.27 ± 1.73 ^a^	4.20 ± 1.28 ^ab^	2.61 ± 0.78 ^abc^

^a^ significantly different compared to the measurement pre-test, *p* < 0.05; ^b^ significantly different compared to the measurement 5 min post-test, *p* < 0.05; ^c^ significantly different compared to the measurement 15 min post-test, *p* < 0.05.

## Data Availability

Data supporting the findings of this study are available from the corresponding author upon request.
